# Establishment of a Rapid Detection Method for Cadmium Ions via a Specific Cadmium Chelator N-(2-Acetamido)-Iminodiacetic Acid Screened by a Novel Biological Method

**DOI:** 10.3390/foods13172684

**Published:** 2024-08-26

**Authors:** Yali Wang, Wenxue Sun, Tinglin Ma, Joseph Brake, Shuangbo Zhang, Yanke Chen, Jing Li, Xiaobin Wu

**Affiliations:** 1 Development Center of Plant Germplasm Resources, College of Life Sciences, Shanghai Normal University, Shanghai 200234, China; yaw32@yulinu.edu.cn (Y.W.); sunwenxue868@163.com (W.S.); tinglinma@163.com (T.M.); zhangshuangbo428@163.com (S.Z.); 2 Department of Chemistry and Chemical Engineering, Yulin University, Yulin 719000, China; michelle_6868@163.com; 3 Department of Biological Chemistry, University of Michigan, Ann Arbor, MI 48109-0600, USA; joebrake@med.umich.edu; 4 Division of Glycoscience, Department of Chemistry, School of Engineering Sciences in Chemistry, Biotechnology and Health, KTH Royal Institute of Technology, AlbaNova University Centre, SE106 91 Stockholm, Sweden

**Keywords:** cadmium, ADA, VBB, rapid detection, food safety

## Abstract

Heavy metal ions such as cadmium, mercury, lead, and arsenic in the soil cannot be degraded naturally and are absorbed by crops, leading to accumulation in agricultural products, which poses a serious threat to human health. Therefore, establishing a rapid and efficient method for detecting heavy metal ions in agricultural products is of great significance to ensuring the health and safety. In this study, a novel optimized spectrometric method was developed for the rapid and specific colorimetric detection of cadmium ions based on N-(2-Acetamido)-iminodiacetic acid (ADA) and Victoria blue B (VBB) as the chromogenic unit. The safety evaluation of ADA showed extremely low biological toxicity in cultured cells and live animals. The standard curve is y = 0.0212x + 0.1723, R^2^ = 0.9978, and LOD = 0.08 μM (0.018 mg/kg). The liner concentrations detection range of cadmium is 0.1–10 μM. An inexpensive paper strip detection method was developed with a detection limit of 0.2 μM to the naked eye and a detection time of less than 1 min. The method was successfully used to assess the cadmium content of rice, soybean, milk, grape, peach, and cabbage, and the results correlated well with those determined by inductively coupled plasma–mass spectrometry (ICP-MS). Thus, our study demonstrated a novel rapid, safe, and economical method for onsite, real-time detection of cadmium ions in agricultural products.

## 1. Introduction

Cadmium is a non-essential element found in trace amounts in the human body with no known biological function and is considered toxic. Under normal environmental conditions, cadmium does not have an impact on human health, but with the recent increase in the production and use of cadmium in industry, excessive cadmium is discharged into the environment through waste gas, waste water, or waste residue [1,2] Peana. This cadmium is absorbed by food crops, leading to its enrichment in the human body and the onset of cadmium toxicity. Health problems associated with cadmium toxicity include osteoporosis, atrophy, deformation, carcinogenesis, teratogenesis, mutagenesis, and other serious health problems [3,4]. The heavy metal cadmium in food and drinking water is specified in detail in accordance with GB 2762-2022 [5] (Limits of Contaminants in Food under the National Standard for Food Safety), and the limit standard of heavy metal cadmium in agricultural products and food is 0.05 mg/kg. The limit of cadmium in mineral water is as low as 0.003 mg/L.

ADA (N-(2-Acetamido)-iminodiacetic acid) is often used as a biological buffer and is used to separate the metallothionein (MT) subtype [6] through combination with other capillary and sample separation technologies. ADA is also used as chelator based on the selective chelation of metal cations in the active center of metalloenzymes to enrich metalloenzymes including laccase C and horseradish peroxidase.

There are many commonly used methods to detect heavy metals, including inductively coupled plasma–mass spectrometry (ICP-MS) [7], electrochemistry [8], functional nanomaterials [9], biosensors [10], and immunochemistry [11]. While these methods can ensure efficient and accurate measurement of cadmium ion content, the instruments are expensive, the detection time is relatively long, and the experimental conditions are harsh, which make them not suitable for real-time detection and carrying in the field. Therefore, development of a faster and less costly method to monitor cadmium concentrations onsite is needed.

In recent years, various paper-based analysis devices (PADs) have received widespread attention owing to their many attributes. PADs are lightweight, highly portable, low-cost, and easy-to-use, and they offer onsite, real-time detection and multi-functional analysis. According to multiple studies, PADs can successfully detect heavy metal ions in foods and the environment [12,13]. At present, a large part of test paper preparations relies on the combination of small molecule metal chelators with functional nanomaterials, fluorescence detectors, electrochemistry, and other technologies as signal generator tools to produce color changes in paper-based analysis equipment. However, issues with the use of metal chelators, such as their metal specificity, toxicity, and synthesis cost, all pose certain obstacles to the preparation of PADs for detecting trace amounts of heavy metal ions. Therefore, it is particularly crucial to screen for a chelator with high specificity, low toxicity, and low cost.

Through metal-ligand chelation therapy, researchers identified N-(2-acetylamino)-iminodiacetic acid (ADA) as an efficient, economical, and easily available complexing agent for thorium ions and conducted detailed thermodynamic research on the complexation between ADA and thorium by means of potentiometric assay, calorimetry, electrospray ionization–mass spectrometry, and theoretical studies [6]. In addition, relevant studies have also reported the affinity constants of manganese, copper, nickel, zinc, and cobalt with ADA at physiological pH and identified possible structures of various metal chelates in solution based on spectral data [14]. Some monitored the change of the ADA dissociation constant through capacitance coupled with contactless conductivity detection (C^4^D) and studied the pH-dependent behavior of two-amino polycarboxylate, which, if fixed on the surface of the monolithic capillary columns, provides instructions for the formation of amino polycarboxylate complexes with metal ions [15]. However, information about ADA research on cadmium is lacking.

In preliminary studies on metal ion metabolism, it was found that certain *Saccharomyces cerevisiae* gene knockout strains were particularly sensitive to specific heavy metal ions, resulting in the inhabited growth of the knockout strain when exposed to low concentration of metal ions [16,17]. In this study, we evaluated the specific affinity of ADA for different metals by using metal-sensitive yeast knockout strains. *Pca1* is a P1B-type ATPase that plays a key role in cadmium resistance in yeast *S. cerevisiae* by extruding intracellular cadmium [18]. Hence, *Pca1 knockout (pca1Δ)* is extremely sensitive to excess cadmium ions. Other metal-sensitive yeasts *(ace1Δ*, *ftr1Δ*, and *fet3Δ)* and the wild-type yeast strains have been used for the toxicity determination of other heavy metals [19,20]. Based on the characteristic that cadmium-sensitive yeast mutants cannot grow on cadmium-supplemented medium, we added the small molecule ADA to eliminate the growth-inhibitory effect of cadmium, and we confirmed the specific binding ability of ADA to cadmium biologically. We demonstrated for the first time that ADA recovers growth of the cadmium-treated *pca1Δ* strain, while other metal-sensitive strains were not protected by co-treatment of ADA with toxic concentrations of their respective sensitizing heavy metals.

Victoria blue B (VBB) is a triarylmethane dye that has been widely utilized in bacteria and botany and as a nuclear stain in cytology and histochemistry [21,22]. In this research, we used the chelate of ADA-Cd to form a stable compound with iodide ions and then performed an ion association reaction with VBB under a suitable ionization equilibrium state. This results in a blue compound formed in the solution with a color intensity proportional to the cadmium content. In order to maximize the detection intensity of the specific signal, we improved and optimized the reaction condition and time in the system. We could successfully detect the content of cadmium within 1 min and applied the color reaction to paper test strips for onsite detection capability. We conducted a safety assessment of ADA and confirmed it was not toxic in cultured cells and a mouse model. Subsequently, we utilized the optimized spectral analysis method to quantitatively detect cadmium in several agricultural products, and we verified the results with inductively coupled plasma–mass spectrometry. Therefore, we demonstrated a new, rapid, economical, and safe method for the detection of cadmium ions.

## 2. Materials and Methods

### 2.1. Materials

All chemical reagents were obtained from Sigma-Aldrich (Shanghai, China), except special instructions.

### 2.2. Yeast Strains, Culture Media, Chelators, Growth Assays, and FTIR

A haploid control yeast, *S. cerevisiae* strain BY4741, and knockout mutants were purchased from Open Biosystems [23]. And the sensitivity of Cd^2+^, Cu^2+^, Cr^3+^, Pb^2+^, and Hg^2+^ were detected using *pca1Δ*, *ace1Δ*, *ftr1Δ*, *fet3Δ*, and wild-type strains, respectively. We cultured yeast cells in complete culture medium (SC) and added 1.5% agar to the liquid culture medium to make solid culture plates. The yeast strains were cultured at 30 °C, and then, the yeast cell growth was measured [24,25]. The WT or knockout cells were grown overnight in SC media, then re-inoculated (OD_600nm_ = 0.2) in fresh medium and grown to mid-log phase (OD_600_ = 0.8–1.0). After dilution to OD_600_ = 0.1 and 3× serial dilutions in sterilized water, 5 µL of cells were spotted on SC plates supplemented with various amounts of CdCl_2_, CuSO_4_, CrCl_3_, Pb (NO_3_)_2_, and Hg (NO_3_)_2_ with or without ADA or the non-specific metal chelator EDTA. The cells were grown at 30 °C for 2 days before photography. Each test was repeated at least three times using three different colonies to confirm the results. We used infrared spectroscopy to identify chelates of ADA and Cd, and the dried materials were analyzed by FTIR equipment (FTIR-7600, Lambda, Sydney, Australia) in KBr particles in the range of 500–4000 cm^−1^.

### 2.3. Toxicological Testing of ADA

Mature female mice were purchased from (Shanghai, China) Slack Laboratory Animal Co., Ltd. We evaluated the safety of ADA using toxicology experiments to determine whether ADA has potential safety risks to the human body. We used the CCK-8 cell proliferation assay to judge the impact of ADA on the growth of mouse monocyte macrophage RAW264.7 cells. When the cell viability is high, the intracellular metabolites will reduce CCK-8 to colored products, and the color depth is directly proportional to cell proliferation and inversely proportional to cytotoxicity. Briefly, ADA at different specified concentrations (0 mM, 0.01 mM, 0.05 mM, 0.1 mM, 0.2 mM, 0.5 mM, 1 mM, 1.5 mM, and 2 mM) was added into the cell culture medium, and cell viability was detected by the absorbance at 450 nm after 1h. Based on the GBZ/T204.2-2011 [26], the acute oral toxicity of ADA was tested in mice. After the mice were given a one-time treatment of ADA, we monitored health across the next 14 days and detected the blood biomarkers to evaluate the toxicity. For initial screening, we calculated the dosage according to the weight of each mouse (~40 g) and gave 0.2 mL ADA per 10 g of weight by intragastric administration. Then, we observed any immediate toxic reactions for at least 10 min after administration to the mice to ensure the dosage of the next mouse. When three mice survived continuously at the selected dose, we moved to a higher dose. The concentrations 100 mg/kg, 200 mg/kg, and 500 mg/kg were selected as the toxic concentration of ADA tested in the formal experiment. During the 14-day observation period, we regularly monitored mouse weight and collected blood for testing of blood parameters. On the 14th day of the experiment, mice were euthanized after a 12 h fast.

### 2.4. Optimization of the Detection System

Starting from the previously established method [27], we assessed how to optimize the system for cadmium detection. The chelate of ADA-Cd forms a complex with I^-^ in potassium iodide and further forms an ionic association with VBB under the combined action of hydrophobic interaction and electrostatic attraction. VBB is partially protonated under the action of sulfuric acid, and ascorbic acid prevents oxidation of the iodine ions under acidic conditions. ADA has the effect of chelating and buffering, which delays the oxidation rate of the solution and extends the retention time of the color. When ADA is put into the system, the solution forms an ionic equilibrium state, and then, ADA and Cd^2+^ chelate and form ligands with I^-^. Finally, a specific blue complex is formed in the system. In order to enhance the intensity of the UV measurement, we compared the effect of different concentrations of KI (0 mM, 20 mM, 40 mM, 60 mM, 80 mM, 100 mM, and 120 mM), ascorbic acid (0 mM, 2 mM, 4 mM, 6 mM, 8 mM, 10 mM, and 12 mM), VBB (0 μM, 5 μM, 10 μM, 15 μM, 20 μM, 25 μM, 30 μM, and 35 μM), sulfuric acid (0 M, 0.2 M, 0.4 M, 0.6 M, 0.8 M, 1 M), ADA (0 mM, 0.5 mM, 1 mM, 1.5 mM, 2 mM, and 2.5 mM), and time on the reaction system. By adding 10 μM of different metal ions (Cd^2+^, Mg^2+^, Ca^2+^, Zn^2+^, Cu^2+^, Na^2+^, Al^3+^, Cr^3+^, Mn^2+^, and Ni^2+^) to the reaction system, we evaluated the specificity and interference. The alteration in color of the test tubes was monitored visually, and the corresponding absorbances were subsequently measured.

### 2.5. Preparation of the Standard Curve

In order to quantify the color reaction on tubes and test paper, a standard curve was made using the optimized reaction conditions (80 mM KI, 4 mM ascorbate, 15 μΜ VBB, 0.6 M H_2_SO_4_, and 1.5 mM ADA). A series of CdCl_2_ concentrations (0 μΜ, 0.1 μΜ, 0.2 μΜ, 1 μΜ, 2 μΜ, and 10 μΜ) were added into the reaction system and were dropped on the test paper (0.5 cm × 0.5 cm). The solutions were measured at 610 nm by spectrophotometer.

### 2.6. Sample Preparation for Color Reaction and ICP-MS Measurement

The rice, soybean, milk, peach, grape, and cabbage were purchased from the market randomly. (Refer to GB 5009.268-2016 [28] method for the pre-processing operations.) Firstly, 0.12 μg/g cadmium ion standard solution of metal ions was added to 0.5 g of sample. Though nitrification treatment, most of the heavy metal ions in agriculture products were dissolved, and then, the absorbance of the mixture was measured at 610 nm by introducing it into the cadmium ion detection system. For the ICP-MS detection, 10% nitric acid was diluted and sent for detecting. Metal ions were quantified by ICP-MS (Agilent Model 7500cs, Santa Clara, CA, USA). The metal ion content was normalized by sample weight [29,30].

### 2.7. Statistical Analysis

Descriptive analyses are presented as the mean ± S.D. and statistical comparisons of control and experimental groups were performed using Student’s *t*-test. *p* < 0.05 was considered to be significant.

### 2.8. Guidelines for Animal Experimentation

In the experimental process, we adhered to the animal ethical regulations and the 3R principles: to optimize the experimental design and standardize the experimental operation under the premise of guaranteeing the accuracy of data and information, guarantee the quality of animals, standardize the breeding of animals, care for the experimental animals, safeguard the welfare of the animals, and reduce the impact of inhumane procedures on the animals so as to ensure the compliance with the ethical principles of experimental animals.

The mice used in the study were approved by Human Participants Ethics Committee of Shanghai Normal University on 12 July 2024.

## 3. Results

### 3.1. A Novel Biological Assay to Test ADA Binding Specificity

**Figure 1:**
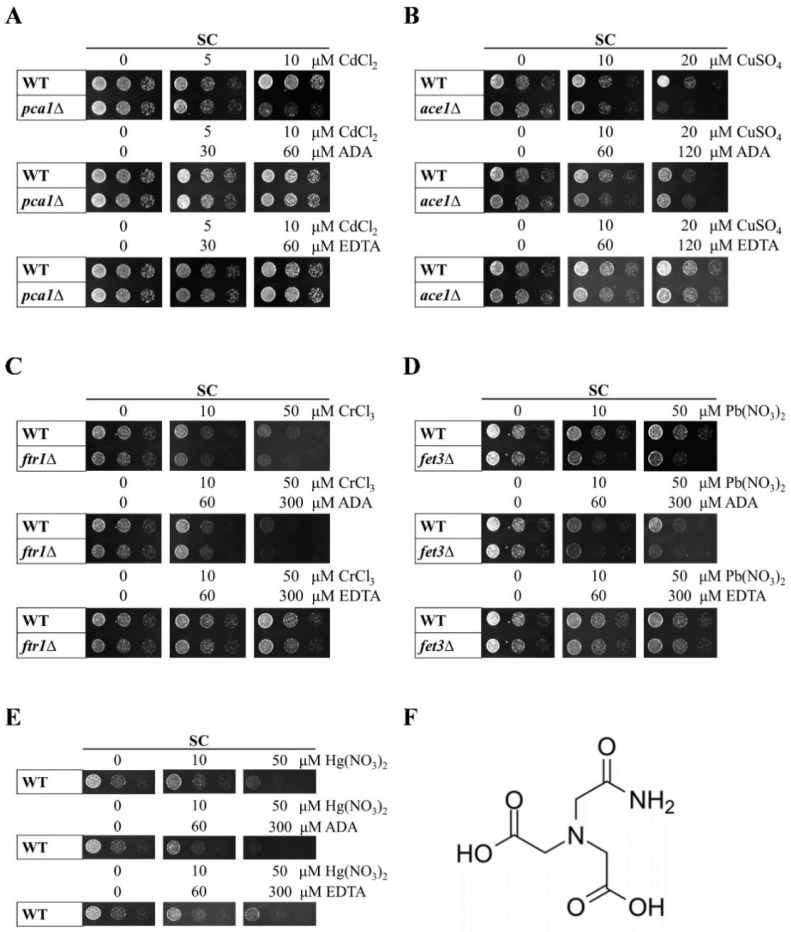
Identification of ADA binding specificity by biological assays. (**A**) Growth of WT control and *pca1Δ* strains in SC solid media containing glucose with CdCl_2_ or ADA or EDTA supplementation at the indicated concentrations. (**B**) Growth of WT control and *ace1Δ* strains in SC solid media containing glucose with CuSO_4_ or ADA or EDTA supplementation at the indicated concentrations. (**C**) Growth of WT control and *ftr1Δ* strains in SC solid media containing glucose with CrCl_3_ or ADA or EDTA supplementation at the indicated concentrations. (**D**) Growth of WT control and *fet3Δ* strains in SC solid media containing glucose with Pb(NO_3_)_2_ or ADA or EDTA supplementation at the indicated concentrations. (**E**) Growth of WT strain in SC solid media containing glucose with Hg(NO_3_)_2_ or ADA or EDTA supplementation at the indicated concentrations. Exponentially growing cells were spotted on media and assessed after 2 days. All growth assays were conducted with at least three different clones. (**F**) Schematic structure of ADA.

**Figure 2:**
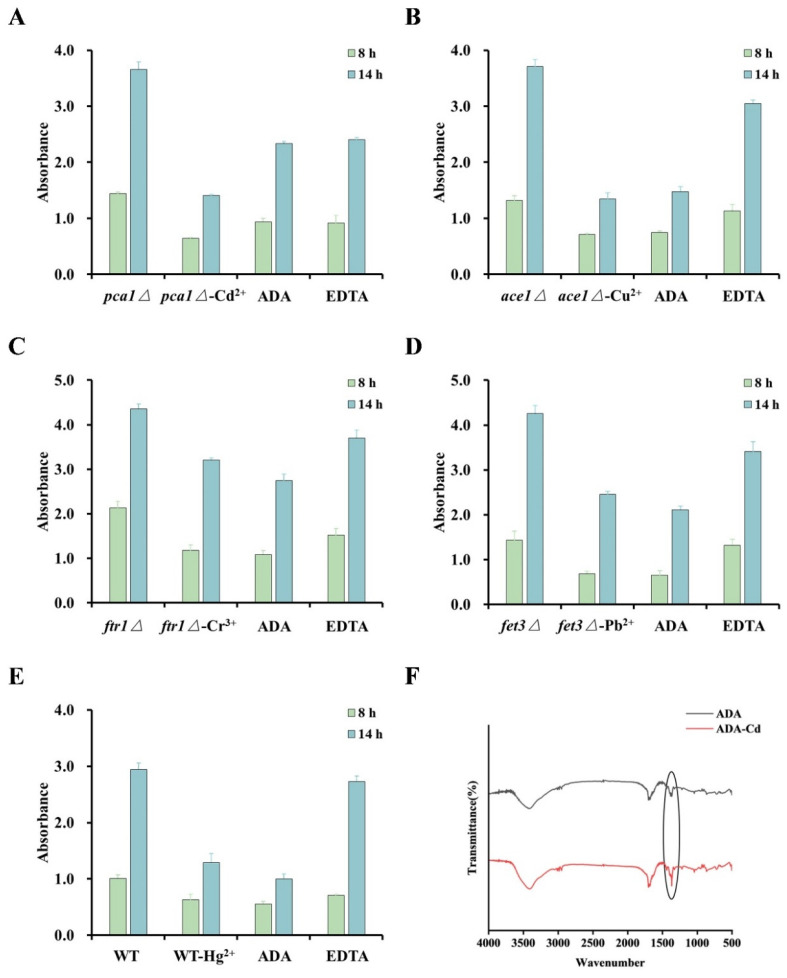
Identification of ADA binding capacity by biological and chemical assays. (**A**) Growth of *pca1Δ* strain in SC liquid media containing glucose with CdCl_2_ or ADA or EDTA supplementation at the indicated concentrations. (**B**) Growth of *ace1Δ* strain in SC liquid media containing glucose with CuSO_4_ or ADA or EDTA supplementation at the indicated concentrations. (**C**) Growth of *ftr1Δ* strain in SC liquid media containing glucose with CrCl_3_ or ADA or EDTA supplementation at the indicated concentrations. (**D**) Growth of *fet3Δ* strain in SC liquid media containing glucose with Pb(NO_3_)_2_ or ADA or EDTA supplementation at the indicated concentrations. (**E**) Growth of WT strain in SC liquid media containing glucose with Hg(NO_3_)_2_ or ADA or EDTA supplementation at the indicated concentrations. (**F**) FTIR spectra of ADA and ADA-Cd.

The high specificity of heavy metal ion chelators is key to developing rapid detection systems for heavy metal ions. Thus, it is important to select a screening method to identify a chelator that meets this requirement. We established a biological detection method based on the sensitivity of different yeast mutants to specific heavy metal ions. In order to ensure the specificity of ADA for cadmium, we evaluated whether ADA could restore the metal-induced toxicity in some metal-sensitive yeast knockout strains. We used the non-specific metal chelator EDTA as a positive control, and the sensitivities of Cd^2+^, Cu^2+^, Cr^3+^, Pb^2+^, and Hg^2+^ were detected using *pca1Δ*, *ace1Δ*, *ftr1Δ*, *fet3Δ*, and wild-type yeast, respectively (Figure 1). The WT and knockout mutants were cultured to mid-log phase, and cells were added to SC solid media with or without ADA or EDTA at different concentrations of CdCl_2_, CuSO_4_, CrCl_3_, Pb(NO_3_)_2_, and Hg(NO_3_)_2_. As we predicted, *pca1Δ* cells were highly sensitive to CdCl_2_ (10 μM), and the growth defect was restored by adding ADA or EDTA (Figure 1A). When other metal-sensitive yeast strains were treated with different toxic metal concentrations, ADA did not restore the growth defects, although they were restored by the EDTA controls (Figure 1B–E). We also detected the effect on the liquid culture growth of each metal within their respective sensitive strains, and the results were consistent with the solid growth experiments (Figure 2A–E). The recovery of cell growth by positive control EDTA in all metal-treated conditions demonstrated that the growth defects were indeed caused by metal toxicity. These results indicated that the metal chelation of ADA was specific for cadmium ions and had no effect on other metal ions. We further identified ADA and its chelates by using infrared spectroscopy to confirm the chelating ability of ADA and cadmium (Figure 2F), which proved the accuracy and effectiveness of the biological method.

### 3.2. Safety Assessment of ADA

**Figure 3:**
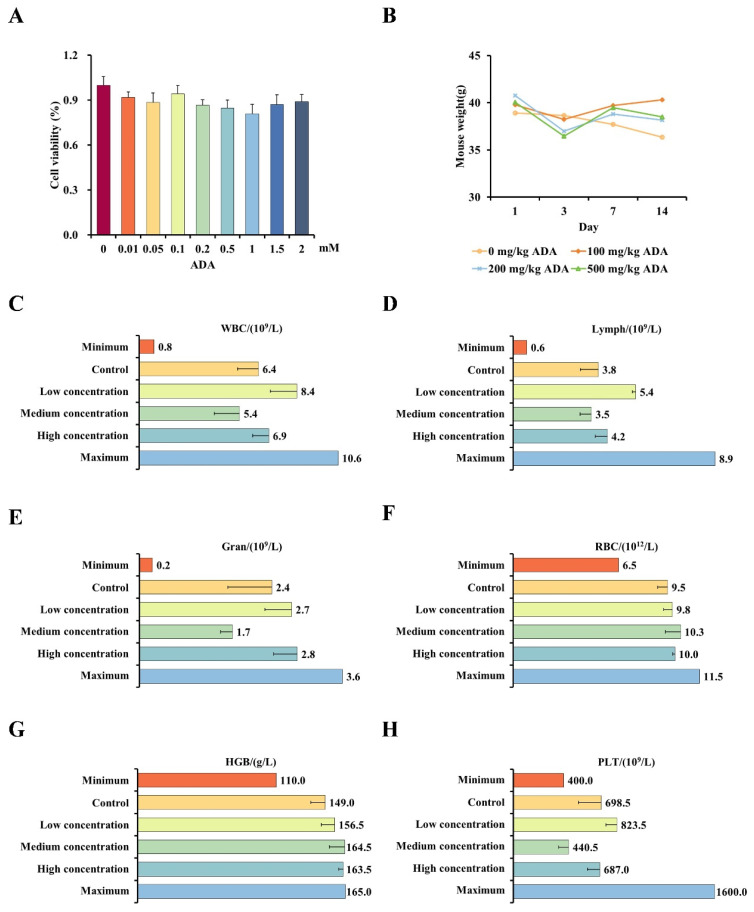
Safety Assessment of ADA. (**A**) Specified concentrations of ADA were added to the culture medium of mouse macrophage RAW 264.7 cells and cell viability was measured by CCK-8 assay. (**B**) Changes in body weight of mice during the observation period. Changes in mouse WBC (**C**), mouse lymph (**D**), mouse gran (**E**), mouse RBC (**F**), mouse HGB (**G**), and mouse PLT (**H**) after adding specified concentrations of ADA. Minimum: minimum value of this indicator in the normal physiological state; Maximum: maximum value of this indicator in the normal physiological state; Control: 0 mg/kg ADA; Low concentration: 100 mg/kg ADA; Medium concentration: 200 mg/kg ADA; High concentration: 500 mg/kg ADA.

We explored the relationship between ADA dose and its biological toxicity at the cellular and organismal levels to confirm the harmful effects of ADA on the body. At the cellular level, increasing the dose of ADA from 0.01 to 2 mM had minimal effect on cell viability (Figure 3A), indicating that ADA has negligible toxic effect on cells. To assess the toxicity of ADA in an animal model, we administered three doses of ADA to mice, namely 100 mg/kg (low), 200 mg/kg (medium), and 500 mg/kg (high), and we observed the markers of animal health over the subsequent 2 weeks. After administration, the mice were supplied a normal diet and drinking water, and there were no signs of acute poisoning or death. We noted no significant change in body weight for 2 weeks after ADA administration (Figure 3B). We conducted blood tests on the mice and found that the white blood cells (WBC), lymphocytes (Lymph), neutrophils (Gran), red blood cells (RBC), hemoglobin (HGB), and platelets (PLT) of the ADA-administered mice were within the normal physiological range (Figure 3C–H). These results indicated that ADA has no toxic effect on mice in the concentration of 0–500 mg/kg. In summary, ADA has low biological toxicity to cells and mice, which suggests it can be safely applied in the detection systems.

### 3.3. Identification of Optimal Reaction Conditions

**Figure 4:**
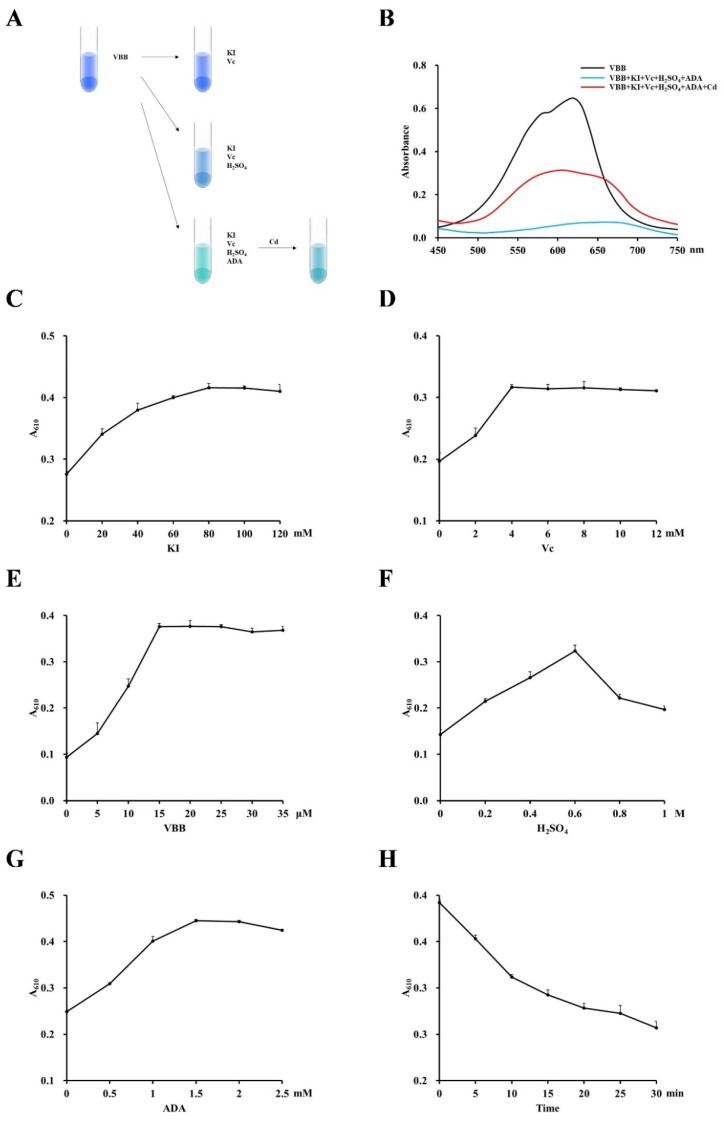
Schematic color change (created with Macros of PowerPoint 2010) of cadmium ions (**A**) and UV spectra (**B**). Optimization of conditions for complex formation. (**C**) The effect of KI on the assay (ascorbate: 4 mM, VBB: 15 μΜ, H_2_SO_4_: 0.6 M, ADA: 1.5 mM, and CdCl_2_: 10 μΜ). (**D**) The effect of ascorbate on the assay (KI: 80 mM, VBB: 15 μΜ, H_2_SO_4_: 0.6 M, ADA: 1.5 mM, and CdCl_2_: 10 μΜ). (**E**) The effect of VBB on the assay (KI: 80 mM, ascorbate: 4 mM, H_2_SO_4_: 0.6 M, ADA: 1.5 mM, and CdCl_2_: 10 μΜ). (**F**) The effect of H_2_SO_4_ on the assay (KI: 80 mM, ascorbate: 4 mM, VBB: 15 μΜ, ADA: 1.5 mM, and CdCl_2_: 10 μΜ). (**G**) The effect of ADA on the assay (KI: 80 mM, ascorbate: 4 mM, VBB: 15 μΜ, H_2_SO_4_: 0.6 M, and CdCl_2_: 10 μΜ). (**H**) The effect of time on the assay (KI: 80 mM, ascorbate: 4 mM, VBB: 15 μΜ, H_2_SO_4_: 0.6 M, ADA: 1.5 mM, and CdCl_2_: 10 μΜ). All assays were conducted at least three times. Average ± S.D. is presented.

The colorimetric response of the conjugate is diagrammed in Figure 4A. We observed maximum absorbance at 610 nm using a spectrophotometer to scan the complex from 450 to 750 nm (Figure 4B). To find the optimal reaction conditions, we screened varying concentrations of KI, ascorbate, VBB, H_2_SO_4_, and ADA and assessed different assay times. Under the condition of 10 μΜ CdCl_2_ with other reaction conditions unchanged, the absorbance at 610 nm was the highest when the concentration of KI reached 80 mM (Figure 4C). Ascorbate reached optimal absorbance in the range of 4–12 mM (Figure 4D). When the VBB concentration reached 15 μM, the absorbance was the highest, and it remained stable at higher concentrations (Figure 4E). The absorbance peak of H_2_SO_4_ was at 0.6 M (Figure 4F). The best effect of ADA was at a concentration of 1.5 mM (Figure 4G). Next, we assessed the stability of the reaction color over time. The blue complex gradually disappeared over time, as seen by the declining absorbance (Figure 4H). Therefore, in order to ensure the accuracy of the results of real-time detection, the detection should be completed within 1 min. Based on the above results, we selected the optimal reaction conditions for the detection system, which consisted of 80 mM KI, 4 mM ascorbate, 15 μΜ VBB, 0.6 M H_2_SO_4_, and 1.5 mM ADA.

### 3.4. Spectrum Method for the Detection of Cadmium

**Figure 5:**
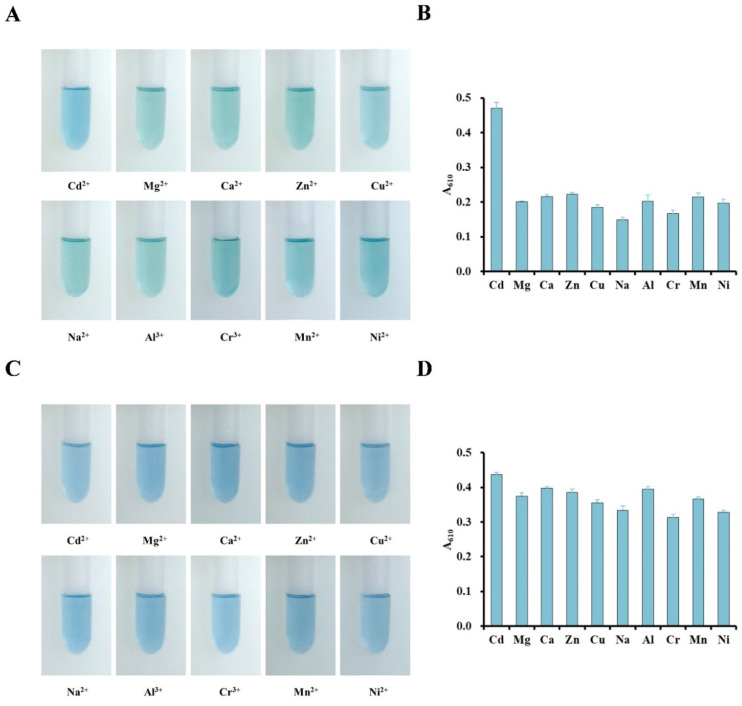
Metal specificity assay and interference assay across other metal ions (Cd^2+^, Mg^2+^, Ca^2+^, Zn^2+^, Cu^2+^, Na^2+^, Al^3+^, Cr^3+^, Mn^2+^, and Ni^2+^), where 10 μM indicated metal ion was added into the system, and solutions were (**A**) imaged and (**B**) quantitated by absorbance at 610 nm. Then, 10 μM indicated metal ion was mixed with Cd^2+^ in the system, and solutions were (**C**) imaged and (**D**) quantitated by absorbance at 610 nm.

**Figure 6:**
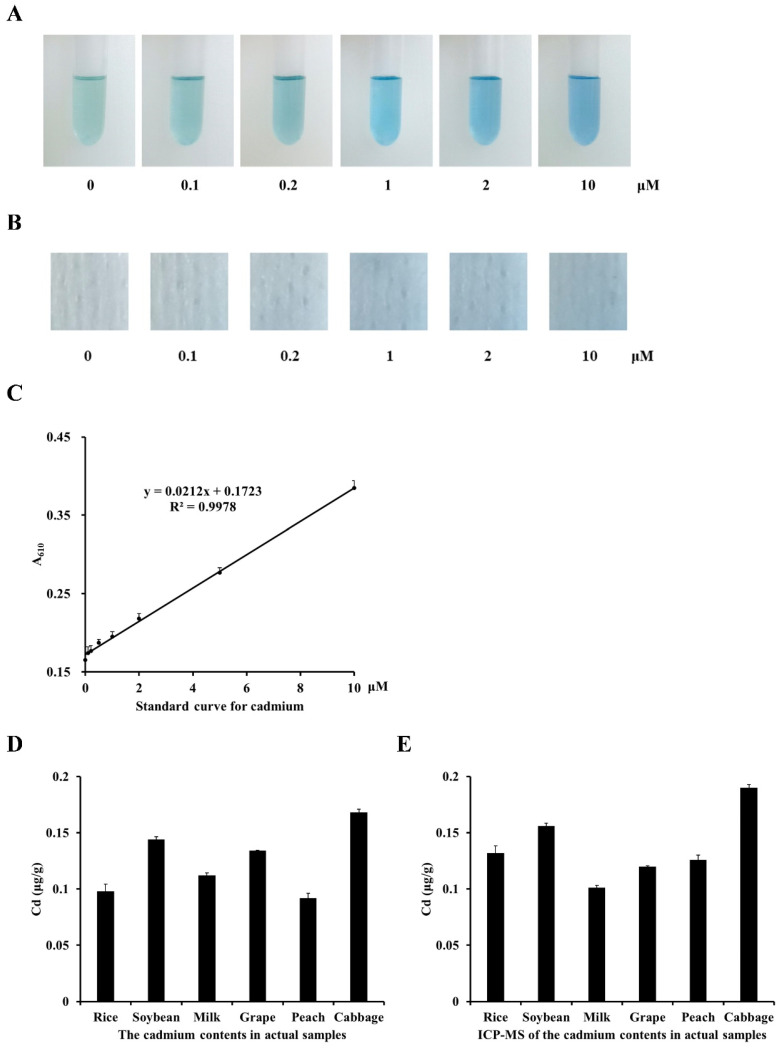
Preparations of standard paper strips and standard curve. Standard solutions were prepared under optimized reaction conditions (KI: 80 mM, ascorbate: 4 mM, VBB: 15 μΜ, H_2_SO_4_: 0.6 M, and ADA: 1.5 mM) with varying concentrations of CdCl_2_ (0, 0.1, 0.2, 1, 2, and 10 μM). (**A**) Standard solutions were imaged in glass test tubes. (**B**) Paper strips (filter paper, 0.5 × 0.5 cm) were soaked in standard solutions, dried for 1 min, and visualized against a white background. (**C**) A standard curve was prepared by measuring the absorbance at 610 nm over Cd concentration. Cd contents of indicated samples were determined by (**D**) optimized spectrum method and (**E**) ICP-MS.

In order to explore the specific recognition ability of the detection system for Cd^2+^, the best reaction system (80 mM KI, 4 mM ascorbate, 15 μΜ VBB, 0.6 M H_2_SO_4_, and 1.5 mM ADA) and 10 μM of different metal ions (Cd^2+^, Mg^2+^, Ca^2+^, Zn^2+^, Cu^2+^, Na^2+^, Al^3+^, Cr^3+^, Mn^2+^, and Ni^2+^) were mixed, and the color change was observed by measuring absorbance of test tube at 610 nm. We found only Cd^2+^ can react with the detection system, while the other ions did not affect the color (Figure 5A) and had no significant impact on absorption (Figure 5B). The results confirmed the system had good selectivity for the detection of Cd^2+^. Next, we analyzed whether other metals could interfere with the detection of Cd^2+^ by co-treating the system with Cd^2+^ and other metals (Cd^2+^, Mg^2+^, Ca^2+^, Zn^2+^, Cu^2+^, Na^2+^, Al^3+^, Cr^3+^, Mn^2+^, and Ni^2+^) under optimal conditions. Imaging of the test tube (Figure 5C) and absorbance (Figure 5D) showed that the detection of Cd^2+^ was not affected by the presence other metal ions. The results indicated that the detection system has good specificity and selectivity for Cd^2+^. Next, we tested whether the absorbance of the complex at 610 nm is proportional to the content of cadmium. To obtain the relationship between reaction color and concentration of cadmium, the optimal reaction system was mixed with CdCl_2_ at 0–10 μM (Figure 6A), and we observed that the color of solution deepens with increasing cadmium concentration. We could observe a color change by as low as 0.2 μM Cd (Figure 6A). We applied the solutions to test papers and dried for 1 min to show the color (Figure 6B). The color deepened with increasing cadmium concentration, similar to the solutions, which demonstrated the viability of the paper strip test method. We established the standard curve of Cd^2+^ at 610 nm (y = 0.0212x + 0.1723, R^2^ = 0.9978) and found the LOD to be 0.08 μM (0.018 mg/kg) and the linear detection range between 0.1 and 10 μM (Figure 6C). The results showed that we successfully established an optimized spectroscopic method with a detection limit of 0.2 μM (0.046 mg/kg) for rapid detection of Cd^2+^.

### 3.5. Cadmium Measurements of Agricultural Products by Optimized Spectrum Method

In order to assess the practical application of the method, we purchased rice, soybean, milk, grapes, peaches, and cabbage randomly from the market. Samples of 0.5 g each were weighed and pre-treated according to the national standard GB 5009.268-2016 [28]. We added metal standard solutions into the samples, and the solutions were added to the reaction system to measure the absorbance at 610 nm. The detection results of rice, soybean, milk, grape, peach, and cabbage by the optimized spectrum method at 610 nm were 0.098 μg/g, 0.144 μg/g, 0.112 μg/g, 0.134 μg/g, 0.092 μg/g, and 0.168 μg/g, respectively (Figure 6D). The ICP-MS results showed that the cadmium contents were 0.132 μg/g, 0.156 μg/g, 0.101 μg/g, 0.12 μg/g, 0.126 μg/g, and 0.19 μg/g, respectively (Figure 6E). Table 1 better demonstrates the reliability of the optimized spectral method. There is a good correlation between the results of ICP-MS and the optimized spectrometry. These results showed that the optimized spectroscopic method can reliably quantify cadmium in agricultural products.

## 4. Discussion

In nature, cadmium mainly exists as cadmium sulfide ore, and a lesser amount also exists in zinc ore. With the development of mineral resources, cadmium pollution has increased in the environment, which is from the waste water discharged by electroplating, mining, and chemical industries. The water polluted by cadmium is seriously harmful to the body, and its detoxification is slow. Hence, it is essential to develop a rapid and efficient method to detect cadmium in the environment and food to ensure human well-being and the safety of the environment.

The commonly used detection methods for Cd^2+^ are atomic fluorescence spectrometry [31,32,33], atomic absorption spectrophotometry [34,35], anodic stripping voltammetry [36,37,38]. These methods are complex and difficult to monitor in real time. In recent years, paper-based analysis devices have entered the public due to their simplicity, speed, and low cost, and they can conduct qualitative or semi-quantitative identification of heavy metals through specific color reactions. In preparing the reaction system, it is important to select chelators with high specificity, low toxicity, and strong chelating ability. First, Cd^2+^ was detected using the chelating and buffering properties of ADA. In order enhance the color of the final complex, we used VBB as the color development unit.

The specificity, interference, and safety of detection systems are the keys to their practical applications. In the study, we utilized yeast mutants, which are sensitive to heavy metals, to identify the binding ability of ADA to various metals. The method showed that ADA has a strong binding capacity for Cd^2+^ but no other metals. We confirmed the safety of ADA through testing its toxicity at the cellular and organismal levels, which is essential to the subsequent establishment of the rapid detection system to reduce harm to the technical operator and to comply with safety standards during the testing process. Before the testing of the agricultural samples, we screened a variety of metals to identify the specificity and interference of Cd^2+^ and analyzed and judged the color and absorbance of the reaction solution. We found that our system has the characteristics of high sensitivity, high specificity, and low interference for the detection of Cd^2+^.

We analyzed the characteristics of sensitivity, specificity, detection time, instrument assistance, detection objects, and cost to compare the method we established with other methods (Table 2). Compared to others, our method has the advantages of high sensitivity, simplicity, onsite operation, and cost efficiency.

The Cd contents of rice, soybeans, milk, grapes, peaches, and cabbage were successfully detected using our optimized spectrometry method, and the results were comparable to those determined by inductively coupled plasma–mass spectrometry. However, our method has some areas that can be improved. First, concentrated acid is preferred for pre-treatment of raw materials due to its high purity and extensive oxidation capacity [48,49,50,51]. Despite the high efficiency of concentrated nitric acid, its application for sample digestion cannot meet the requirements of green analytical chemistry. For this study, we used the national method for sample pre-treatment; however, we will improve the concentrated acid treatment conditions to develop more effective strategies. Lee et al. studied diluted nitic acid and hydrogen peroxide mixtures as another way to optimize acid digestion while minimizing environmental impact [52].

Second, we need to improve the detection limit by improving the color of intensity under low Cd^2+^ concentrations. Nanomaterials have unique chemical properties that show a wide range of application in improving detection sensitivity. Chen et al. used DL-mercaptosuccinic acid-modified gold nanoparticles to detect Cd^2+^, with a detection limit of 0.7 mM [40]. Noh et al. utilized a protein-directed method to synthesize fluorescent cadmium nanoclusters for sensitive and selective detection of Cd^2+^, with a detection limit of 15.68 fM [53]. Guo et al. designed a sample non-labeled colorimetric method using unmodified gold nanoparticles and glutathione in a high-salt environment to detect Cd^2+^ in water and rice with a visual detection line of 10 μM [47]. Tao et al [54]. established a colorimetric method using MoS_2_ nanocomposites modified with gold nanoparticles as an enzyme mimetic to detect Cd^2+^ with a detection limit of 0.7 ng/mL [55].

Third, it is worth improving the quality of the test paper. In this study, the test paper used is an ordinary color-absorbing cloth purchased in the market, which can produce a reasonable color detection reaction. We will explore using higher-quality test paper to increase the intensity of the color reaction. In order to avoid subjectivity in color judgement, a phone may be used to detect the color change, which would be practical for rapid onsite analysis [56,57].

Our results demonstrated a new detection system for environmental Cd^2+^ that presents a color change in an aqueous reaction system and on test paper that is proportional to Cd^2+^ content. We successfully applied our method to determine the cadmium content in agricultural products, namely fruits and vegetables. Our system is, fast, cost-effective, safe, and practical for onsite application.

## Figures and Tables

**Table 1:** Optimized spectrum method compared with ICP-MS ion.

	Method	Concentration (μg/g)	RSD (%)	Adding Standard Recovery (%)
Rice	OSM	0.098	4.8	70
	ICP-MS	0.132	4.7	98
Bean	OSM	0.144	2.5	103
	ICP-MS	0.156	1.6	113
Milk	OSM	0.112	3.3	90
	ICP-MS	0.101	2.2	81
Grape	OSM	0.134	1.3	106
	ICP-MS	0.12	0.5	94
Peach	OSM	0.092	3.8	72
	ICP-MS	0.126	3.2	100
Cabbage	OSM	0.168	1.1	120
	ICP-MS	0.19	1.5	138

Note: OSM: optimized spectrum method.

**Table 2:** Comparison of rapid detection methods for cadmium ion.

Method	Sensitivity	Specificity	Detection Time	Instrument Aids	Detection Object	Cost	Reference
This method	0.2 μM	high	1 min	No	fruits, vegetables, agricultural products	Lower	
Colorimetric	0.53 μM	high	2 min	No	water	Low	[39]
Nanomaterial	70 μM	good	Not given	No	aqueous solutions	Low	[40]
Fluorescence	0.15 μM	good	90 min	Yes	aqueous solutions	Medium	[41]
biosensor	0.5 μM	medium	Not given	Yes	water	Medium	[42]
Nanomaterial	0.23 μM	high	10 min	No	water	Low	[43]
Nanomaterial	20 μM	good	30 min	No	aqueous solutions	Low	[44]
Nanomaterial	0.24 μM	high	15 min	No	water	Low	[45]
Nanomaterial	0.24 μM	high	70 min	Yes	water	Medium	[46]
Nanomaterial	10 μM	high	10 min	No	rice	Low	[47]

## Data Availability

The original contributions presented in the study are included in the article, further inquiries can be directed to the corresponding authors.

## Abbreviations

ADA	N-(2-Acetamido)-iminodiacetic acid
VBB	Victoria blue B
ICP-MS	inductively coupled plasma–mass spectrometry
MT	metallothionein
PADs	paper-based analysis devices
WBC	white blood cells
Lymph	lymphocytes
Gran	neutrophils
RBC	red blood cells
HGB	hemoglobin
PLT	platelets
